# Nonrandomized Open Trial of a Mindfulness- and Compassion-Based Selective Preventive Mobile Health Intervention for Forcibly Displaced People

**DOI:** 10.1007/s12671-025-02675-9

**Published:** 2025-10-02

**Authors:** Shani Zohar Puris, Kim Yuval, Gebreyohans Gebremariam, Simon B. Goldberg, Noga Aviad, Scott A. Baldwin, Amit Bernstein

**Affiliations:** 1 Department of Psychology, School of Psychological Sciences, University of Haifa, Haifa, Israel; 2 Department of Counseling Psychology, School of Education, University of Wisconsin–Madison, Madison, United States; 3 Department of Psychology, Brigham Young University, Utah, United States; 4 Center for Healthy Minds, College of Letters and Science, University of Wisconsin–Madison, Madison, United States

**Keywords:** Forcibly displaced people, Mindfulness- and compassion-based intervention, Mobile health, COVID-19

## Abstract

**Objectives:**

This study tested the feasibility of Mindfulness-SOS for Refugees, a novel lay- and minimally guided mobile health mindfulness- and compassion-based intervention that is trauma-sensitive and socio-culturally adapted for diverse forcibly displaced people.

**Method:**

A pre-registered, nonrandomized, single-arm, open trial of Mindfulness-SOS as a selective preventive intervention was conducted during the COVID-19 pandemic among 60 Eritrean asylum seekers living in an unstable urban post-displacement setting in the Middle East (Israel). Measures included digital usage metrics and self-report measures of stress- and trauma-related mental health and socio-contextual stressors.

**Results:**

Asylum seekers (*n* = 58) demonstrated high rates of adherence to the session modules and generally moderate rates of overall adherence. Elevated pre-intervention post-traumatic stress symptoms severity and post-migration living difficulties stressors prospectively predicted lower levels of engagement with meditation practice exercises. Finally, greater engagement with meditation practice exercises was associated with attenuated deterioration in depression and anxiety, but not with change in post-traumatic stress symptoms, from pre- to post-intervention.

**Conclusions:**

Mindfulness-SOS may be a feasible selective preventive intervention approach among asylum seekers in stressful post-displacement settings.

**Preregistration:**

The study was preregistered at ClinicalTrials.gov (NCT04761510; clinicaltrials.gov; 2021-02-17).

**Supplementary Information:**

The online version contains supplementary material available at 10.1007/s12671-025-02675-9.

Over 120 million people are currently forcibly displaced by conflict, persecution, and natural disasters (United Nations High Commissioner for Refugees [UNHCR], 2024). Forcibly displaced people (FDP), among them refugees and asylum seekers, often struggle with stress- and trauma-related mental health conditions (Blackmore et al., 2020; Bogic et al., 2015; Priebe et al., 2016). Moreover, multiple barriers impede FDP’s access to mental health care post-displacement (Byrow et al., 2020; Kiselev et al., 2020; Satinsky et al., 2019). Accordingly, a portfolio of mental health interventions tailored for FDP communities is needed (Bajbouj et al., 2021; Grasser, 2022; Silove, 2021). Fortunately, substantial progress in intervention science dedicated to FDP mental health has emerged over the past decade (Kronick et al., 2021).

Mindfulness-based (Aizik-Reebs et al., 2021; Neslihan, 2021; Van der Gucht et al., 2019) and mindfulness-informed (Blignault et al., 2021; Foka et al., 2021; Shaw et al., 2019; Tol et al., 2020; Tubbs Dolan et al., 2021) interventions tailored to FDP represent one promising therapeutic approach within this portfolio (Jeebodh-Desai & Dwarika, 2022; Pillay & Eagle, 2019; Sun et al., 2022). Moreover, in a review of 44 meta-analyses of randomized controlled trials, mindfulness-based interventions demonstrated effects comparable to or better than evidence-based active controls on depression, depressive relapse, stress, or other psychiatric conditions or symptoms among diverse populations (Goldberg et al., 2022b), though primarily Western populations and contexts (Carlson, 2018; DeLuca et al., 2018; Sun et al., 2022; Waldron et al., 2018).

The rationale for a mindfulness-based intervention approach to FDP mental health is multi-faceted (Aizik-Reebs et al., 2021). First, mindfulness training has been linked to reduced severity, persistence, and recurrence of chronic stress-related symptoms (Creswell, 2017; Goldberg et al., 2022b). Second, flexible delivery formats for mindfulness-based interventions are well-suited to help address implementation barriers faced by FDP mental health intervention efforts. For example, mindfulness-based interventions target processes that transcend language and culture, such as awareness and compassion; they are typically brief, group-based, can be delivered and practiced through various modalities (e.g., mobile-based), and may be guided by remotely supervised lay people from within the target community (Aizik-Reebs et al., 2021; Pillay & Eagle, 2019). These interventions can also be adapted to be trauma-sensitive to ensure their safety and efficacy among trauma-affected populations such as FDP (Jeebodh-Desai & Dwarika, 2022; Treleaven, 2018; Waelde, 2022), and may be socio-culturally adapted to suit diverse marginalized populations and contexts (Loucks et al., 2022).

Initial research has begun to test the feasibility and therapeutic efficacy of mindfulness- and compassion-based and mindfulness-informed intervention programs among FDP. Aizik-Reebs et al. (2021) conducted a waitlist controlled trial of Mindfulness-Based Trauma Recovery for Refugees (MBTR-R), among a trauma-affected community sample of East African asylum seekers (*n* = 158) residing in an urban post-displacement setting in the Middle East (Israel) (Aizik-Reebs et al., 2021, 2022a, 2022b; Amir et al., 2023; Oren-Schwartz et al., 2023). MBTR-R demonstrated acceptability, feasibility, safety, and significant improvement in stress- and trauma-related mental health outcomes at post-intervention and 1-month follow-up assessments relative to a waitlist control group. Furthermore, randomized controlled trials and pilot studies of mindfulness-informed group-based interventions among diverse FDP subgroups and communities (Epping-Jordan et al., 2016; Hinton et al., 2004, 2005) have demonstrated initial, albeit mixed, evidence of acceptability, feasibility (Eskici et al., 2023; Kananian et al., 2017; Tol et al., 2018a, 2018b), and treatment and/or preventive efficacy (Acarturk et al., 2022; Augustinavicius et al., 2023; Eskici et al., 2023; Kananian et al., 2020; Purgato et al., 2021; Turrini et al., 2022).

The fact that these interventions are delivered in-person limits their access, reach, and scalability (Kazdin, 2017; Schick et al., 2018). For FDP, barriers to implementation are exacerbated by the complex, unstable, and low-resourced post-displacement settings wherein FDP increasingly reside, making mental health care inaccessible for most (Al-Soleiti et al., 2021; Mutiso et al., 2018). Thus, behavioral intervention technologies (BITs) (i.e., psycho-behavioral intervention strategies that employ a range of technologies), and specifically, mobile health (mHealth) technologies such as mobile apps or web-based tools (Mohr et al., 2013), could help improve access to evidence-based mental health care for FDP (El-Haj-Mohamad et al., 2023; El-Refaay et al., 2024; Liem et al., 2021; Mabil-Atem et al., 2024; Sijbrandij et al., 2017). Mindfulness-based and mindfulness-informed BITs and mHealth technologies are well-suited to expand access, reach, scalability, feasibility, flexibility, and potential cost-effectiveness of such interventions (Gal et al., 2021; Goldberg et al., 2022a; Linardon, 2020; Mrazek et al., 2019; Tan et al., 2022). Meta-analyses have demonstrated their efficacy for mental health outcomes among Western populations and contexts, with small-to-moderate therapeutic effects on depression and anxiety symptoms (Gal et al., 2021; Linardon et al., 2024; Sommers-Spijkerman et al., 2021; Tan et al., 2022).

The World Health Organization (World Health Organization [WHO], 2017) has pioneered the adaptation of evidence-based mental health care for FDP to scalable BITs and mHealth technologies. For example, Step-by-Step is a cognitive-behavioral therapy-based, lay-guided self-help intervention for depression, based on Behavioral Activation. It is delivered through a culturally adapted BIT- or mHealth-based modularized and personalized five-session intervention program (Carswell et al., 2018). In a randomized controlled trial in Lebanon among Syrian FDP, minimally guided Step-by-Step demonstrated promising efficacy post-intervention for stress- and trauma-related mental health conditions, impaired functioning, and well-being, relative to usual care (Cuijpers et al., 2022). Although Step-by-Step was locally adapted (Abi Ramia et al., 2018; Burchert et al., 2018) and showed largely positive results in randomized controlled trials and pilot studies with regard to feasibility and acceptability (i.e., among intervention completers), adherence to the intervention program was low (e.g., in a fully powered two-armed randomized clinical trial, 32.2% of participants completed all sessions) (Cuijpers et al., 2022; Harper Shehadeh et al., 2019; Heim et al., 2021).

Indeed, adherence and engagement are a central challenge to the utility of BITs and mHealth broadly (Baumel et al., 2019; Koh et al., 2022; Linardon, 2020) and mindfulness-based and mindfulness-informed BITs, specifically (Linardon, 2023; Liptáková et al., 2022; Osborne et al., 2023; Winter et al., 2022). Knowledge of factors that predict or interfere with their adherence and engagement is limited and inconclusive (Liptáková et al., 2022; Osborne et al., 2023). Moreover, candidate predictors of BITs for FDP remain largely undocumented (Abi Ramia et al., 2023). As mindfulness-based interventions are skill-based, adherence and engagement with training over time may be therapeutically vital (Creswell, 2017; Goldberg et al., 2025; Parsons et al., 2017). One strategy to address these challenges has focused on incorporating guidance, in varying formats and levels of intensity, into the design and delivery of mindfulness-based BITs (Osborne et al., 2023; Sommers-Spijkerman et al., 2021; Spijkerman et al., 2016; Zhang et al., 2020).

We thus focus here on three key gaps in extant intervention science, and specifically mindfulness- and compassion-based interventions, tailored to FDP. First, a mindfulness- and/or compassion-based mHealth intervention tailored to FDP has yet to be developed or tested. Second, to optimize and personalize the development and delivery of BITs for FDP (Abi Ramia et al., 2023), and mindfulness-based and mindfulness-informed BITs more specifically (Liptáková et al., 2022; Osborne et al., 2023), we need greater knowledge of barriers to BITs adherence, engagement, feasibility, and therapeutic efficacy among FDP (Augustinavicius et al., 2023; Cuijpers et al., 2022; El-Haj-Mohamad et al., 2023; El-Refaay et al., 2024; Liem et al., 2021; Mabil-Atem et al., 2024). Further, no study has been conducted among FDP testing dose-response effects of mindfulness meditation training broadly or their BITs applications specifically. Prior to the conduct of randomized control trial research (Onken et al., 2014), such knowledge is necessary to understand the forms and degree of adherence and engagement with mindfulness- and compassion-based BITs required for therapeutic effects among FDP.

The overarching aim of this study was to conduct an initial, pre-registered, nonrandomized, single-arm, open trial to explore the feasibility of Mindfulness-SOS for Refugees. Mindfulness-SOS is a novel mindfulness- and compassion-based mHealth intervention program tailored to diverse FDP, implemented with lay- minimally guided support. The principles, content, and meditation practice exercises of Mindfulness-SOS were based closely on the group-based MBTR-R intervention program (Aizik-Reebs et al., 2021). Mindfulness-SOS was tested here as a selective prevention intervention (Munoz et al., 1996) among trauma-affected asylum seekers, due to its implementation during an acute lock-down phase at the peak of the COVID-19 crisis, in order to prevent mental health deterioration due to acute pandemic-related stress (Blay Benzaken et al., 2023; El Tatary & Gill, 2022; Kiteki et al., 2022; Liddell et al., 2021; Sevim et al., 2023).

Aim 1 was to estimate Mindfulness-SOS feasibility by digitally monitoring and calculating objective participant-level metrics of adherence and engagement with intervention session modules and meditation practice exercises. Aim 2 was to test the prospective associations between pre-intervention factors (e.g., stress- and trauma-related mental health, socio-contextual stressors, demographics) and degree of Mindfulness-SOS adherence and engagement. Aim 3 was first, to test the effect of time, from pre- to post-intervention, on stress- and trauma-related mental health outcomes, and second, to test the protective dose-response effects of Mindfulness-SOS adherence and engagement by estimating the associations between levels of adherence and engagement and stress- and trauma-related mental health outcomes post-intervention.

## Method

### Participants

This study was a nonrandomized, single-arm, selective preventive intervention open trial of Mindfulness-SOS for Refugees (Mindfulness-SOS). The study was conducted among a community sample of Eritrean asylum seekers residing in an urban post-displacement setting in the Middle East (Israel) (*M* = 35.69, *SD* = 4.58 years; 50% (*n* = 30) female). The trial was conducted between September 2020 and January 2021, during the COVID-19 pandemic national lockdowns. The selected population of Eritrean asylum seekers is representative of a large and fast-growing proportion of FDP worldwide (United Nations High Commissioner for Refugees [UNHCR], 2024). This is a high-risk population of asylum seekers experiencing high levels of stress- and trauma-related mental health difficulties, associated with high exposure to traumatic experiences and chronic stress (e.g., Aizik-Reebs et al., 2022b; Bachem et al., 2024). During the study trial, COVID-19 control policies coupled with pre-existing migration and residential status policies led to acute and significant deterioration of stress- and trauma-related mental health conditions among this community and post-displacement setting (Blay Benzaken et al., 2023). This selective preventive intervention program was thus used to buffer mental health deterioration among this at-risk population.

Over the course of three successive months, participants were recruited from the community via public flyers, social networks, and local non-governmental and municipal organizations working with FDP. Exclusion criteria were (a) active suicidality (i.e., active plan to commit suicide and/or past suicide attempt and/or passive suicidal ideation with clinical indicators of imminent suicide risk); (b) current mental health treatment (i.e., psychotherapy or/and group therapy at least twice a month); and (c) previous participation in a Mindfulness-Based Trauma Recovery for Refugees (MBTR-R) group.

Ninety-six adult Eritrean asylum seekers were recruited and screened for participation. Sixty participants consented and qualified for inclusion and were then assigned to the intervention and completed the pre-intervention assessment. Fifty-nine participants (98%) completed the post-intervention assessment. For more details on participants, see Fig. 1 and Online Resource 1.

### Procedure

The study was conducted within an ambulatory laboratory temporarily housed in a non-governmental organization dedicated to the rights of FDP, located in the urban center of this asylum seekers’ community. To ensure health and safety, COVID-19 social distancing and health regulations were strictly adhered to during in-person meetings. Lay cultural mediators assisted with communication between participants and research staff.

Following informed consent in participants’ first language (Tigrinya), participants completed a self-report pre-intervention assessment. A suicidality risk assessment was performed prior to study registration. Eligible participants received a username/password to access the digital intervention program and then listened to the first Mindfulness-SOS session module together with the lay cultural mediator and research staff. Technical guidance was provided during this launch meeting, in addition to brief, illustrated tutorials. Participants were provided with headphones to ensure they could listen to the intervention program privately.

Participants were instructed to listen to the Mindfulness-SOS session modules and meditation practice exercises over a 4-week period. Participants were encouraged to listen to 2 session modules per week, complemented by meditation practice exercises that are paired with each session module. Thus, participants were encouraged to practice mindfulness meditation at least once daily, and to listen to at least two session modules per week, in a pre-determined order. To maximize access and adherence, participants were able to utilize the intervention program at any time or place of their choosing. To optimize therapeutic impact, participants were encouraged to engage with Mindfulness-SOS in as quiet an environment as possible. To ensure maximal personalization and flexibility of utilization, we permitted participants up to 8 weeks to complete the intervention program. Upon completion of the mHealth program or following 8 weeks, participants returned to the ambulatory laboratory in-person to complete a post-intervention assessment. Follow-up assessments were logistically not feasible in this unstable high-risk urban post-displacement setting during the COVID-19 crisis.

#### Mindfulness-SOS for Refugees: Intervention Program

Mindfulness-SOS is a novel mHealth adaptation of MBTR-R, a manualized mindfulness- and compassion-based group intervention that is trauma-sensitive and socio-culturally adapted for diverse populations of FDP (See Aizik-Reebs et al., 2021 for details). Mindfulness-SOS entails audio 8 brief training session modules (each 7–38 min/session) and 10 complimentary audio meditation practice exercises (each 4–25 min/practice), delivered in Tigrinya using participants’ smartphones. Relative to MBTR-R, Mindfulness-SOS session modules and some of the meditation practice exercises are shorter in duration, mobile (e.g., not scheduled, not in-person, not led by instructor), and not delivered in group-based format (i.e., no group-based learning, practice, or inquiry).

The therapeutic principles, components, and meditation practice exercises of Mindfulness-SOS are based on the group-based MBTR-R program and adapted to a mHealth format. Like MBTR-R, Mindfulness-SOS entails each of the following key components: (1) **experiential learning through mindfulness meditation practice exercises**, including systematic training in formal meditation practice exercises (e.g., body scan, mindful movement, sitting meditation) and informal meditation practice exercises (e.g., mindful awareness in daily living); as well as loving-kindness and/or self-compassion practice exercises to counter maladaptive self-referentiality such as shame and self-criticism (Aizik-Reebs et al., 2022a; Aizik-Reebs et al., 2021; van den Brink & Koster, 2015). (2) **Psychoeducation** to facilitate understanding of stress and trauma, to normalize and de-stigmatize common mental health difficulties, and to normalize difficulties in mindfulness meditation training. (3) Trauma-sensitive adaptations (e.g., safe place adaptation of mindfulness meditation, language that invites to facilitate participant-directed choice and control) to help ensure safety and potential to benefit from meditation practice exercises among trauma-affected participants. (4) **Socio-cultural adaptations** (e.g., linguistic adaptation, integration of metaphors and idioms to communicate key principles) to facilitate delivery of intervention program principles and meditation practice exercises, and thereby optimize intervention program efficacy among diverse populations of FDP (Bhambhani & Gallo, 2022; Harper Shehadeh et al., 2016; Sobczak & West, 2013). (5) **Modeling key salutary attitudes and qualities of mindfulness** (e.g., patience, acceptance, non-judging, curiosity, or beginner’s mind, letting go, and kindness) through session modules and meditation practice exercises.

Like the group-based MBTR-R, the mHealth Mindfulness-SOS intervention program is delivered through a sequence of scaffolded therapeutic elements, as follows: (1) intervention program orientation; (2) normalization and destigmatization; (3) psychoeducation about trauma, chronic and acute stress, as well as attention; (4) trauma-sensitive readiness training for meditation (e.g., practice and cultivation of a safe place practice exercise to support subsequent meditation practice exercises, communicating choice and agency); (5) introduction to principles and attitudes of mindfulness; (6) mindful movement practice exercise; (7) focused attention meditation; (8) mindfulness of difficult and pleasant experiences, and challenges during meditation; (9) psychoeducation on nurturing versus depleting activities; (10) self-compassion training and practice exercise; (11) self-compassion practice exercise in response to difficulties; and (12) integrating mindfulness principles and practice exercises into daily life.

In addition, to further ensure the safety of the intervention program and meditation practice exercises among trauma-affected participants, Mindfulness-SOS did not include MBTR-R session modules or meditation practice exercises designed to elicit exposure to conditioned trauma responses (Aizik-Reebs et al., 2021). Conditioned trauma responses may be associated with overwhelming distress, maladaptive trauma coping mechanisms, and difficulties in remaining engaged with training, which might require more intensive guidance (i.e., beyond the current phone-based minimal-guidance from lay cultural mediators) (Kearney & Simpson, 2020a, 2020b).

#### Minimally Guided Mindfulness-SOS (MG-Mindfulness-SOS)

To support engagement with Mindfulness-SOS, participants received guidance via weekly phone calls and text messages, personalized to their past-week program utilization. This included guide-initiated administrative guidance (e.g., technical guidance in case of mobile- or internet platform-related difficulties, and information about the intervention program or assessment), as well as adherence-focused guidance (e.g., adherence monitoring including weekly brief check-in phone calls and text-based nudges to guide, remind and prompt adherence) (Leung et al., 2022; Moshe et al., 2021; Saleem et al., 2021). To facilitate scalability, phone-based minimal-guidance, in Tigrinya, was manualized, standardized, and delivered by lay cultural mediators. Lay cultural mediators received structured training in the Mindfulness-SOS manual, the phone-based minimal-guidance delivery format, and risk management protocols (e.g., suicidality risk). A psychologist with expertise in the intervention program trained and supervised the mediators.

### Measures

#### Linguistic and Cultural Translation Procedure

All measures were administered in Tigrinya, following translation and back-translation in accordance with structured guidelines (Brislin, 1970; Geisinger, 1994), and in partnership with professional translators, FDP mental health researchers, service providers working with this asylum-seeker community, and lay cultural mediators from this community. All translated measures were pilot-tested and revised in an iterative process, including cognitive interviewing, with cultural mediators and other asylum seekers, to ensure linguistic as well as socio-cultural meaning. Measures were psychometrically evaluated and validated in multiple earlier studies (Aizik-Reebs et al., 2021; Yuval et al., 2021).

#### Stress and Trauma-Related Mental Health Outcomes

The Harvard Trauma Questionnaire (HTQ; Mollica et al., 1992) was used to measure traumatic stress exposure. The Harvard Trauma Questionnaire-5 (HTQ-5; Berthold et al., 2019) was used to measure symptom severity of Post-Traumatic Stress (PTS) (according to DSM-V criteria) (*α* = 0.94, *ωt* = 0.95). The nine-item Patient Health Questionnaire (PHQ-9; Spitzer et al., 1999) was used to measure symptom severity of depression (*α* = 0.87, *ωt* = 0.87). A brief version of the Beck Anxiety Inventory (BAI; Beck et al., 1988), which included 6 items that loaded most strongly on the total BAI scores in previous studies among this population (Aizik-Reebs et al., 2021), was used to measure symptom severity of anxiety (*α* = 0.90, *ωt* = 0.89).

#### Candidate Socio-contextual Stressors as Predictors of Intervention Program Engagement

The Post-Migration Living Difficulties scale (PMLD; Silove et al., 1997) was used to measure post-migration living difficulties stressors (*α* = 0.80, *ωt* = 0.79). The COVID-19 Socioeconomic Insecurity Index (Blay Benzaken et al., 2023) was used to measure the impact of the pandemic on housing (e.g., Did your housing change because of COVID-19?), food (e.g., Did you lose access to food because of COVID-19?), and income (e.g., Did your employment change because of COVID-19?) security.

#### Mindfulness-SOS Intervention Program Utilization: Digital Objective Metrics

We measured participant-level metrics of behavioral adherence and engagement with Mindfulness-SOS using objective digitally monitored log data of intervention program utilization. Adherence was quantified as the number of the prescribed session modules and meditation practice exercises completed by participants, with a maximum of 8 session modules and 10 meditation practice exercises. Engagement was quantified as the total number of session modules and/or meditation practice exercises completed by participants; as participants were able to listen to individual session modules and meditation practice exercises repeatedly. Incomplete Sessions + Practices was quantified as the relative percentage of session modules and/or meditation practice exercises that were initiated but not completed (either terminated or interrupted). Finally, self-guided intervention duration was quantified as the total number of weeks participants engaged with the intervention program, up to 8 weeks.

### Data Analyses

All analyses were conducted among the intent-to-treat sample, excluding two participants with outlier engagement values from the analyses (*n* = 58). Results were not changed when including these two participants in a sensitivity analysis. See Fig. 1 in Online Resource 1.

For analyses of Aims 2 and 3: (a) Limited variability in adherence (ceiling effect) precluded the use of adherence as a predictor or outcome in analyses; (b) a critical *p*-value of 0.05 for all statistical tests was necessary to mitigate high risk for type II errors due to small sample, with small-to-medium expected effect sizes (Fiedler et al., 2012); and (c) we reported the results of regression analyses for both session modules and meditation practice exercises, respectively. Additional analyses of combined session modules and practice exercises were not included, in order to limit unnecessary inflation of family-wise alpha.

#### Aim 1. Mindfulness-SOS Feasibility

To estimate feasibility, we calculated participant-level metrics of behavioral intervention program adherence, engagement, incomplete sessions + practices, and self-guided intervention duration.

#### Aim 2. Predictors of Mindfulness-SOS Engagement

Ordinary least squares (OLS) regression was used to examine whether and to what degree candidate pre-intervention factors (i.e., stress- and trauma-related mental health, socio-contextual stressors, demographics) prospectively predicted levels of (1) engagement with session modules and/or meditation practice exercises and (2) the relative percentage of incomplete session modules and/or meditation practice exercises.

#### Aim 3. Protective Dose-Response Effects of Mindfulness-SOS Engagement

First, a paired *t*-test was used to estimate pre- to post-intervention changes for stress- and trauma-related mental health outcomes (i.e., PTS, depression, anxiety symptoms). Second, an OLS regression was conducted to estimate dose-response associations between levels of engagement and residualized change scores from pre- to post-intervention for stress- and trauma-related mental health outcomes. In addition, the same analytic approach was applied to examine associations between the relative percentage of incomplete session modules and/or meditation practice exercises and outcomes.

## Results

### Sample and Context: Descriptive Statistics

Participants were Eritrean asylum seekers, 27 to 47 years old (*M* = 35.69, *SD* = 4.58), 50% were women, and their education levels varied between 1 and 6 years (23.73%), 7 and 12 years (62.71%), and 13 and 16 years (13.56%). At pre-intervention, participants reported extensive trauma history, including exposure to multiple traumatic event types (*M* = 6.36, *SD* = 3.70). Likewise, participants reported severely elevated levels of post-migration living difficulties stress (*M* = 3.26, *SD* = 0.98). Participants reported high levels of COVID-19-related socioeconomic insecurity, including food, housing, and income insecurity (*M* = 2.00, *SD* = 0.92) reported forms of COVID-19-related insecurity (possible range of 0 to 4). Finally, participants reported elevated mean levels of PTS symptoms (*M* = 2.12, *SD* = 0.67), with 50% of participants exceeding the threshold for clinically severe levels of symptoms (i.e., HTQ-5 $\ge$ 2). Participants also reported elevated levels of depression symptoms (*M* = 9.28, *SD* = 6.52), with 42.37% exceeding the threshold for clinically moderate levels of symptoms (i.e., PHQ-9 ≥ 10). Additionally, participants reported elevated levels of anxiety symptoms (*M* = 1.16, *SD* = 0.84), with 31.48% reporting moderate or severe levels on three or more of six symptoms. See Table 1 for descriptive statistics of the intent-to-treat sample and post-migration context.

**Table 1:** Descriptive statistics of sample and post-migration context and intervention effects among the intent-to-treat sample

Demographic characteristics							
	Pre-intervention assessment						
	*n*	*M *(*SD*)					
Age	58	35.69 (4.58)					
Education	59	1.90 (0.61)					
Trauma history (HTQ)	59	6.36 (3.70)					
Post-Migration Living Difficulties (PMLD)	59	3.26 (0.98)					
COVID-19-related socioeconomic insecurity	60	2.00 (0.92)					
Stress- and trauma-related mental health outcomes							
	Pre-intervention assessment		Post-intervention assessment		*t*	*p*	*d*
	*n*	*M *(*SD*)	*n*	*M *(*SD*)			
Post-traumatic stress symptoms (HTQ-5)	57	2.12 (0.67)	57	2.18 (0.59)	1.02	0.31	0.14
Depression symptoms (PHQ-9)	58	9.28 (6.52)	58	8.22 (5.68)	−1.49	0.14	−0.20
Anxiety symptoms (BAI)	58	1.16 (0.84)	58	1.27 (0.79)	1.25	0.22	0.16
*Note.HTQ*, Harvard Trauma Questionnaire (Mollica et al., 1992); *PMLD*, Post-Migration Living Difficulties scale (Silove et al., 1997); *HTQ-5*, Harvard Trauma Questionnaire-5 (Berthold et al., 2019); *PHQ-9*, Nine-item Patient Health Questionnaire (Spitzer et al., 1999); *BAI*, a six-item version of the Beck Anxiety Inventory (Beck et al., 1988)							

### Aim 1. Mindfulness-SOS Feasibility

See Table 2 for descriptive statistics of the participant-level metrics of intervention program utilization (*n* = 58).

**Table 2:** Participant-level metrics of intervention program utilization

	Session modules		Meditation practice exercises		Session modules + meditation practice exercises	
	*n*	*M *(*SD*)	*n*	*M *(*SD*)	*n*	*M *(*SD*)
Adherence	58	7.34 (1.74)	58	5.40 (3.73)		
Engagement	58	12.71 (6.39)	58	8.52 (6.76)	58	21.22 (10.47)
Total Number of Incomplete Sessions + Practices	57	6.30 (4.44)	57	2.53 (2.12)	57	8.82 (5.20)
Relative Percentage of Incomplete Sessions + Practices (%)	57	32% (*SD* = 16%)	51	29% (*SD* = 24%)	57	30% (*SD* = 15%)

*Note*. Adherence, the number of prescribed session modules and meditation practice exercises completed by participants, from a total of 8 session modules and 10 meditation practice exercises; Engagement, the total number of session modules and/or meditation practice exercises completed by participants; Total Number of Incomplete Sessions + Practices, the total number of initiated session modules and meditation practice exercises that were never complete; Relative Percentage of Incomplete Sessions + Practices (%), the relative percentage of session modules and meditation practice exercises that were initiated but not completed

#### Adherence

Eighty-five percent (*n* = 49) of participants completed all 8 prescribed session modules (*M* = 7.34, *SD* = 1.74). Fifty-five percent (*n* = 32) of participants completed more than half (i.e., ≥ 6) of the 10 prescribed meditation practice exercises (*M* = 5.40, *SD* = 3.73), and 15.52% (*n* = 9) of participants did not engage in any of the meditation practice exercises.

#### Engagement

Participants completed a mean total of 12.71 session modules (*SD* = 6.39) and 8.52 meditation practice exercises (*SD* = 6.76). The degree of engagement with session modules and meditation practice exercises was significantly, although modestly, positively correlated (*r*(56) = 0.27, *p* = 0.04).

#### Incomplete Sessions + Practices

Participants completed, on average, 68% (*SD* = 16%) of all initiated session modules, and 71% (*SD* = 24%) of all initiated meditation practice exercises; thus, 32% of session modules and 29% of meditation practice exercises, respectively, were initiated but not completed. More specifically, participants initiated but did not complete a mean total of 6.30 (*SD* = 4.44) session modules and 2.53 (*SD* = 2.12) meditation practice exercises.

#### Self-guided Intervention Duration

The intervention program was completed by participants over an average of 4.33 weeks (*SD* = 1.62), with 56.90% (*n* = 33) of participants completing it within 3 to 4 weeks, 34.48% (*n* = 20) within 5–8 weeks, and 8.62% (*n* = 5) within 2 weeks.

### Aim 2. Predictors of Mindfulness-SOS Engagement

Greater pre-intervention PTS symptoms severity (*F*_(1,54)_ = 6.96, *β* = − 0.34, *p* = 0.01, ${R}^{2}$ = 0.11), and levels of post-migration living difficulties (*F*_(1,55)_ = 4.85, *β* = − 0.29, *p* = 0.03, ${R}^{2}$ = 0.08), each significantly predicted lower levels of engagement with meditation practice exercises. Depression and anxiety symptoms severity, sex assigned at birth, and COVID-19-related socioeconomic insecurity did not predict levels of engagement with meditation practice exercises. In contrast to engagement with meditation practice exercises, no pre-intervention factor predicted levels of engagement with session modules.

We also examined whether these candidate barriers to engagement were prospectively associated with the relative percentage of incomplete session modules or meditation practice exercises. Women were not significantly more likely to initiate but to not complete session modules, however, the effect size despite the null effect may be noteworthy (*F*_(1,55)_ = 3.50, *β* = 0.24, *p* = 0.07, ${R}^{2}$ = 0.06). Thus, no pre-intervention factor was significantly related to the relative percentage of incomplete session modules or meditation practice exercises. See Table 3 for regression statistics of levels of prospective pre-intervention factors predicting engagement with session modules or mediation practice exercises. Also, see Table 5 in Online Resource 1 for regression statistics of levels of prospective pre-intervention factors predicting the relative percentage of incomplete sessions + practices levels (*n* = 58).

**Table 3:** Linear regression of levels of prospective pre-intervention factors predicting engagement

Predictors	Engagement with session modules					Engagement with meditation practice exercises				
	*F*	*df*	*β*	*p*	*R*^2^	*F*	*df*	*β*	*p*	*R*^2^
Sex assigned at birth	0.22	56	0.06	0.64	0.00	0.15	56	−0.05	0.70	0.00
Post-Migration Living Difficulties (PMLD)	1.41	55	0.16	0.24	0.02	4.85 *	55	−0.29	0.03	0.08
COVID-19-related socioeconomic insecurity	0.47	56	0.09	0.49	0.01	3.89	56	−0.26	0.05	0.07
Post-traumatic stress symptoms (HTQ-5)	0.20	54	−0.06	0.66	0.00	6.96 **	54	−0.34	0.01	0.11
Depression symptoms (PHQ-9)	0.00	55	−0.00	1.00	0.00	0.02	55	0.02	0.90	0.00
Anxiety symptoms (BAI)	0.15	55	−0.05	0.70	0.00	1.82	55	−0.18	0.18	0.03

*Note.* Asterisks *, **, and *** represent significance levels at 0.05, 0.01, and 0.001, respectively

*PMLD*, Post-Migration Living Difficulties scale (Silove et al., 1997); *HTQ-5*, Harvard Trauma Questionnaire-5 (Berthold et al., 2019); *PHQ-9*, Nine-item Patient Health Questionnaire (Spitzer et al., 1999); *BAI*, a brief version of the Beck Anxiety Inventory (Beck et al., 1988)

### Aim 3. Protective Dose-Response Effects of Mindfulness-SOS Engagement

#### Pre-to-Post-Intervention Effects

First, there was no significant change in PTS (*t*(56) = 1.02, *p* = 0.31; *d* = 0.14), depression (*t*(57) = −1.49, *p* = 0.14; *d* = −0.20), or anxiety (*t*(57) = 1.25, *p* = 0.22; *d* = 0.16) symptoms from pre-to-post intervention. See Table 1 for descriptive statistics of the pre-to-post-intervention effects of the intent-to-treat sample.

#### Protective Dose-Response Effects of Mindfulness-SOS Engagement

Second, greater engagement with meditation practice exercises significantly predicted less deterioration in depression (*F*_(1,54)_ = 5.22, *β* = −0.30, *p* = 0.03, ${R}^{2}$ = 0.09) and in anxiety (*F*_(1,54)_ = 4.68, *β* = −0.28, *p* = 0.04, ${R}^{2}$ = 0.08) symptoms, but not in PTS symptoms, from pre- to post-intervention. Neither levels of engagement with session modules, nor the relative percentage of incomplete session modules or meditation practice exercises predicted the degree of symptom change, from pre- to post-intervention, in PTS, depression, or anxiety symptoms outcomes. See Table 4 for regression analyses statistics of levels of engagement predicting levels of residualized change scores of stress- and trauma-related mental health outcomes. Also, see Table 6 in Online Resource 1 for regression analyses statistics of levels of relative percentage of incomplete sessions + practices predicting levels of residualized change scores of stress- and trauma-related mental health outcomes (*n* = 58).

**Table 4:** Linear regression of levels of engagement predicting levels of residualized change scores of stress- and trauma-related mental health outcomes

Predictors	Engagement with session modules					Engagement with meditation practice exercises				
	*F*	*df*	*β*	*p*	*R*^2^	*F*	*df*	*β*	*p*	*R*^2^
Post-traumatic stress symptoms (HTQ-5)	0.04	53	−0.03	0.84	0.00	0.20	53	0.06	0.66	0.00
Depression symptoms (PHQ-9)	3.61	54	−0.25	0.06	0.06	5.22 *	54	−0.30	0.03	0.09
Anxiety symptoms (BAI)	0.74	54	−0.12	0.39	0.01	4.68 *	54	−0.28	0.04	0.08

*Note.* Asterisks *, **, and *** represent significance levels at 0.05, 0.01, and 0.001, respectively

*HTQ-5*, Harvard Trauma Questionnaire-5 (Berthold et al., 2019); *PHQ-9*, nine-item Patient Health Questionnaire (Spitzer et al., 1999); *BAI*, a brief version of the Beck Anxiety Inventory (Beck et al., 1988)

## Discussion

The present nonrandomized, single-arm, open trial tested the feasibility of Mindfulness-SOS, a novel mindfulness- and compassion-based mHealth intervention program, as a selective preventive intervention for FDP, at the height of the COVID-19 pandemic. Due to pandemic control measures, which curtailed the already limited access and reach of care among marginalized communities at risk, the mHealth delivery format and test was critical and timely (Benjamen et al., 2021; Fauk et al., 2021; Leung et al., 2023).

We found initial evidence supporting the feasibility of Mindfulness-SOS among this asylum-seeker community and post-displacement setting. Using objective utilization metrics, we found that asylum seekers demonstrated high rates of adherence to the session modules and generally moderate rates of overall adherence. This pattern of utilization was more pronounced for intervention session modules (85% completed all 8 sessions) than for the meditation practice exercises (55.17% completed more than 5 of 10 meditation practice exercises). Relative to a systematic review of adherence to mindfulness-based BITs in non-clinical populations and clinical populations (Liptáková et al., 2022), the observed levels of adherence — especially for session modules — were high.

It is also notable that asylum seekers did not complete 32% of all initiated session modules and 29% of meditation practice exercises. Perhaps this type of a more refined utilization metric provides a quantitative proxy of barriers (e.g., intrapersonal, environmental), despite motivation to engage (Alshurafa et al., 2018). Future research may examine whether such utilization metrics may serve as a quantitative proxy for barriers to an effective and meaningful engagement with BITs, above and beyond common engagement metrics (Baumel, 2022; Yardley et al., 2016). This knowledge is important for BITs adherence and engagement, optimization and personalization (Perski et al., 2017; Yardley et al., 2016), or for more temporally and contextually sensitive intervention delivery (Hardeman et al., 2019; Nahum-Shani et al., 2015, 2022).

One possible account for the observed high rates of adherence to the session modules may relate to the minimal-guidance delivery format of Mindfulness-SOS. Indeed, evidence suggests that guidance generally improves the utilization of BITs (Baumeister et al., 2014; Weisel et al., 2019; Werntz et al., 2023), and specifically mindfulness-based and mindfulness-informed BITs (Osborne et al., 2023; Sommers-Spijkerman et al., 2021; Spijkerman et al., 2016; Zhang et al., 2020). Because of the socio-cultural and contextual characteristics of many FDP communities, this type of hybrid, guided BIT delivery format may be particularly important. Barriers to engagement for FDP may include varying levels of digital literacy (Burchert et al., 2018; Spanhel et al., 2022), socio-contextual stressors related to post-migration living difficulties — such as social isolation and disconnection — which could be further intensified by the added burden of pandemic control measures faced by FDP (Byrow et al., 2022; Trew et al., 2023), as well as significant mental health difficulties (El-Refaay et al., 2024). Although important initial work using guided BITs designed for FDP is emerging (Cuijpers et al., 2023; El-Haj-Mohamad et al., 2023), the role or optimal forms of guidance in BITs for FDP have not yet been studied systematically.

With respect to candidate barriers to engagement, pre-intervention levels of PTS symptom severity, and post-migration living difficulties, were prospectively associated with lower levels of engagement with meditation practice exercises. Notably, we presume that differential effects for meditation practice exercises but not session modules utilization were due to the ceiling effect of uniformly high levels of adherence for session modules. These findings, linking trauma-related mental health and related post-migration living difficulties with BITs engagement, may inform conditions for feasibility and for optimizing and personalizing Mindfulness-SOS and other BITs for FDP (El-Haj-Mohamad et al., 2023; Goodman et al., 2021; Mabil-Atem et al., 2024). Recent studies have indicated that participants with more severe mental health symptoms (Geurts et al., 2021; Marchand et al., 2019) and lower socioeconomic status (Foale et al., 2024) may be less likely to comply with mindfulness treatments, although these findings are, respectively, mixed and preliminary. Study of such effects with respect to mindfulness-based and mindfulness-informed BIT engagement is needed (e.g., Liptáková et al., 2022; Osborne et al., 2023).

In addition, although not a statistically significant effect, women demonstrated somewhat higher rates of initiated but incomplete session modules than did men. We suggest that, despite motivation in engaging with BITs, women, particularly in this socio-cultural context, face different gender role norms and related life demands (e.g., parenting demands) (Feldman-Savelsberg, 2022; Stewart et al., 2015), which might have functioned as barriers to uninterrupted engagement (Nahum-Shani et al., 2022). We also suggest that potentially higher rates of initiated but incomplete session modules and/or mediation practice exercises among women were paradoxically consistent with evidence that women demonstrate greater levels of BITs adherence and engagement (Borghouts et al., 2021; Lipschitz et al., 2023), as observed in extant mindfulness research (Liptáková et al., 2022). This possibility deserves further study in relation to gender-sensitive BITs optimization and personalization, broadly and for post-displacement contexts specifically.

There was no evidence of group-level preventive effects, from pre-to-post intervention, in PTS, depression, or anxiety symptom outcomes. These null effects on stress- and trauma-related outcomes may be accounted for by evidence that the acute lock-down phase of the COVID-19 pandemic exacerbated pre-existing post-migration living difficulties and mental health vulnerabilities among FDP (Hoffman et al., 2023; Kiteki et al., 2022; Liddell et al., 2021), leading to deterioration in anxiety and depression outcomes, in particular (Blay Benzaken et al., 2023; Camara et al., 2023; Sevim et al., 2023).

Nevertheless, and as may be expected (Cox et al., 2024; Manigault et al., 2021; Parsons et al., 2017), we found protective preventive dose-response effects for depression and anxiety symptom outcomes. Specifically, higher engagement with meditation practice exercises was associated with less observed worsening of depression and anxiety symptom outcomes from pre-to-post intervention. No dose-response effect was observed for PTS symptom outcome, perhaps because PTS may be chronic and tied to past traumatic experiences and their processing (Ehlers & Clark, 2000), and therefore was less exacerbated by the COVID-19 crisis (Akhtar et al., 2021; Ashby et al., 2022), relative to depression and/or anxiety symptom outcomes (Blay Benzaken et al., 2023; Camara et al., 2023; Sevim et al., 2023). Consequently, the protective preventive intervention effects of Mindfulness-SOS may have been more likely to mitigate deterioration of symptoms specifically exacerbated by COVID-19 (Rauschenberg et al., 2021). Furthermore, for safety reasons, the intervention program protocol of Mindfulness-SOS deliberately omitted programming components from the MBTR-R designed to facilitate exposure to conditioned trauma responses (Aizik-Reebs et al., 2021), potentially important therapeutically to enable recovery from PTS (Nemeroff et al., 2006). This choice may have limited the intervention program’s capacity to produce measurable changes in PTS symptom levels within this severely trauma-affected sample. In addition, or alternatively, the therapeutic elements of the intervention program may have lacked sufficient dose or intensity to effectively engage the high severity of trauma-related symptoms in this sample, potentially limiting its impact on PTS symptom outcomes. Nevertheless, the observed dose-response effects for depression and anxiety symptom outcomes align with findings from other studies of mindfulness-based and mindfulness-informed interventions and BITs, conducted primarily in Western populations (Bowles et al., 2023; Goldberg et al., 2025; Linardon, 2023; Strohmaier, 2020; Strohmaier et al., 2020), suggesting that the intervention program may help prevent certain aspects of psychological distress, even under extreme adversity.

### Limitations and Future Directions

This study had several limitations. First, the trial employed a nonrandomized, single-arm, open-trial design without a control group, limiting any capacity for causal inference. As part of a developmental model in intervention science, preliminary open trials are essential for evaluating feasibility and initial signals of intervention promise before proceeding to more resource-intensive randomized controlled trials (Onken et al., 2014). Further, the study was conducted during the acute phase of the COVID-19 pandemic among a high-risk population of FDP living in unstable post-displacement conditions. Under these exceptional circumstances, the implementation of randomization and a controlled comparison group was not logistically viable and posed ethical concerns, particularly in light of the elevated mental health vulnerabilities in this population (Blay Benzaken et al., 2023). Second, although not feasible in the challenging post-displacement context during the COVID-19 crisis, the absence of prospective follow-up data limits understanding of the persistence of observed dose-response effects beyond the immediate post-intervention period. Third, while this study provided initial support for the feasibility of Mindfulness-SOS, modest sample size limited statistical power to detect small but clinically meaningful effects. Accordingly, we did not perform alpha corrections (Fiedler et al., 2012). Consequently, the null effects reported in the study — such as the absence of significant changes in stress- and trauma-related mental health outcomes — should be interpreted with caution, as they likely reflect the preliminary and underpowered nature of this initial feasibility study. This underscores the need for larger, fully powered randomized controlled trials to rigorously assess the efficacy of Mindfulness-SOS. Such trials will be essential for validating the observed dose-response patterns and evaluating symptom change outcomes under more statistically robust conditions. Fourth, this study focused solely on one structured individual guidance delivery format — minimally guided Mindfulness-SOS. Although critical to FDP intervention scalability, a more intensive guidance approach may increase engagement with the intervention program, which may facilitate greater therapeutic gains, yet potentially at the cost of reduced reach and access (Musiat et al., 2022; Shim et al., 2017). Fifth, although the study relied on well-established, socio-culturally adapted self-report measures, structured interviews or clinical ratings were not used in order to avoid potential bias in measuring mental health outcomes (e.g., due to stigma) (Kirmayer et al., 2017; Nickerson et al., 2020; Warner et al., 2011). Sixth, the study was limited to quantitative methods and did not incorporate qualitative or mixed-methods approaches. Although our feasibility assessment included several quantitative indicators, it lacked the depth and contextual insight that participant-centered qualitative data can provide about intervention feasibility and acceptability (Abi Ramia et al., 2023; Bear et al., 2024; O’Keeffe et al., 2019). Such data may be important to identify subjective barriers and opportunities to better facilitate engagement and adherence. Finally, the generalizability of the findings is limited by the specific cultural, geopolitical, and temporal context in which the study was conducted. The study focused on Eritrean asylum seekers living in an unstable urban post-displacement setting in the Middle East (Israel) during the COVID-19 pandemic. As such, the extent to which these findings can be generalized to other FDP populations, different geopolitical environments, or post-pandemic settings deserves further study.

These limitations highlight the need for a larger, rigorous randomized controlled experimental trial to ensure reliable and generalizable results. To do so, we suggest that adaptive intervention approaches (Almirall et al., 2014) could be crucial, particularly for skill-based BITs for FDP that require practice for skill development (i.e., mindfulness meditation). Adaptive intervention designs (e.g., stepped-care) may be particularly important, to not only optimize this and related BITs for FDP, but to develop an efficient and potentially cost-effective public health intervention approach (Jeitani et al., 2024). Accordingly, future trials could explore personalized, adaptive, stepped-care approaches for delivering Mindfulness-SOS, using experimental designs like Sequential Multiple Assignment Randomized Trials (SMART; Clough & Casey, 2015; Collins et al., 2007). Likewise, future research should also investigate the theorized mechanistic role of targeted processes (e.g., self-compassion) (Aizik-Reebs et al., 2022a), as well as more rigorously evaluate dose-response effects using experimental manipulation of dose. Finally, we recommend that future research integrate qualitative or mixed-methods frameworks to more comprehensively evaluate feasibility, acceptability, inform cultural adaptation, and guide implementation of BITs in diverse FDP populations and contexts.

The current preliminary study sought to contribute to global mental health research, digital health and BIT intervention science, intervention science for FDP, and mindfulness- and compassion-based intervention research. We presented preliminary evidence supporting the feasibility of a minimally guided mindfulness- and compassion-based mHealth selective preventive intervention program, tailored to trauma-affected asylum seekers residing in a high-risk post-displacement setting. Likewise, findings may inform ongoing development and optimization of other BITs tailored to FDP. Despite the challenges of conducting clinical research in complex post-displacement settings, efforts to address issues of access and reach of mental health interventions among FDP hold timely and significant relevance for global public health.

## Supplementary Information

Below is the link to the electronic supplementary material.

Supplementary file 1 (DOCX 88.5 KB)

## Data Availability

Investigators can contact authors to access reported de-identified data, materials and/or code.

## Declarations

### Artificial Intelligence

Artificial intelligence was not used.
