# Supplementary material for: Nonrandomized Open Trial of a Mindfulness- and Compassion-Based Selective Preventive Mobile Health Intervention for Forcibly Displaced People
Source: Mindfulness (N Y). 2025 Oct 2;16(10):2845–62. doi: 10.1007/s12671-025-02675-9 (PMC12494159; doi:10.1007/s12671-025-02675-9)
Supplement: Supplementary file 1 — (DOCX 88.5 KB) [file 12671_2025_2675_MOESM1_ESM.docx]

Online Resource 1 Supplemental Materials for “Nonrandomized Open Trial of a Mindfulness- and Compassion-Based Selective Preventive Mobile Health Intervention for Forcibly Displaced People”

Mindfulness

**Method Supplemental**

**Study Design & Participants**

The selected population of Eritrean asylum-seekers represent a large and rapidly growing proportion of Forcibly Displaced People (FDP) living in high-risk urban post-displacement settings worldwide (United Nations High Commissioner for Refugees [UNHCR], 2024). Many members of the Eritrean community were exposed to numerous traumatic experiences pre- and peri-migration (e.g., persecution, torture, human trafficking) (Aizik-Reebs et al., 2021; Connell, 2012; van Reisen et al., 2017; Yuval et al., 2021). Moreover, many members of the selected community are affected by post-migration living difficulties associated with insecure residency status, detention, and deportation (Agur Orgal et al., 2019; Rozen, 2015), as well as chronic daily stressors (Giacco et al., 2018; Li et al., 2016; Miller & Rasmussen, 2017; Yuval et al., 2021). As a result, this community suffers from very high rates (55-80%) of stress- and trauma-related mental health difficulties (Nakash et al., 2017; Yuval & Bernstein, 2017; Yuval et al., 2016).

Notably, two participants were excluded from all relevant analyses due to observed values of engagement outliers, with session modules and meditation practice exercises which were > 3 *SD*s above the mean. In a post-intervention interview, these two participants reported that they repeatedly played the intervention audio session modules and practice exercises in the background while working, but were not necessarily engaging with the session modules or meditation practice exercises as prescribed. Thus, this form of utilization did not permit us to use the digital utilization metrics for these two subjects. See Figure 1 for more details on the flow of participants through each stage of the study trial.

**References**

Agur Orgal, Y., Liberman, G., & Kook Avivi, S. (2019). Israel’s ‘voluntary’ return policy to expel refugees: The illusion of choice. In *Mobile Africa: Human trafficking and the digital divide* (pp. 209-238). Langaa RPCIG.

Aizik-Reebs, A., Yuval, K., Hadash, Y., Gebreyohans Gebremariam, S., & Bernstein, A. (2021). Mindfulness-Based Trauma Recovery for Refugees (MBTR-R): Randomized waitlist-control evidence of efficacy and safety. *Clinical Psychological Science*, *9*(6), 1164-1184. <https://doi.org/10.1177/2167702621998641>

Beck, A. T., Epstein, N., Brown, G., & Steer, R. A. (1988). An inventory for measuring clinical anxiety: Psychometric properties. *J Consult Clin Psychol*, *56*(6), 893-897. <https://doi.org/10.1037//0022-006x.56.6.893>

Berthold, S. M., Mollica, R. F., Silove, D., Tay, A. K., Lavelle, J., & Lindert, J. (2019). The HTQ-5: Revision of the Harvard Trauma Questionnaire for measuring torture, trauma and DSM-5 PTSD symptoms in refugee populations. *Eur J Public Health*, *29*(3), 468-474. <https://doi.org/10.1093/eurpub/cky256>

Connell, D. (2012). Escaping Eritrea: Why they flee and what they face. *Middle East Report 264 (Fall 2012)*, 2-9. <https://merip.org/2012/08/escaping-eritrea/>

Giacco, D., Laxhman, N., & Priebe, S. (2018). Prevalence of and risk factors for mental disorders in refugees. *Semin Cell Dev Biol*, *77*, 144-152. <https://doi.org/10.1016/j.semcdb.2017.11.030>

Li, S. S., Liddell, B. J., & Nickerson, A. (2016). The relationship between post-migration stress and psychological disorders in refugees and asylum seekers. *Curr Psychiatry Rep*, *18*(9), 82. <https://doi.org/10.1007/s11920-016-0723-0>

Miller, K. E., & Rasmussen, A. (2017). The mental health of civilians displaced by armed conflict: An ecological model of refugee distress. *Epidemiol Psychiatr Sci*, *26*(2), 129-138. <https://doi.org/10.1017/S2045796016000172>

Nakash, O., Nagar, M., Shoshani, A., & Lurie, I. (2017). The association between perceived social support and posttraumatic stress symptoms among Eritrean and Sudanese male asylum seekers in Israel. *International Journal of Culture and Mental Health*, *10*(3), 261-275. <https://doi.org/10.1080/17542863.2017.1299190>

Rozen, S. (2015). *Deported to the unknown*. Hotline for Refugees and Migrants. <https://hotline.org.il/en/publication/deported-to-the-unknown-2/>

Silove, D., Sinnerbrink, I., Field, A., Manicavasagar, V., & Steel, Z. (1997). Anxiety, depression and PTSD in asylum-seekers: Associations with pre-migration trauma and post-migration stressors. *Br J Psychiatry*, *170*(4), 351-357. <https://doi.org/10.1192/bjp.170.4.351>

Spitzer, R. L., Kroenke, K., & Williams, J. B. (1999). Validation and utility of a self-report version of PRIME-MD: The PHQ primary care study. Primary Care Evaluation of Mental Disorders. Patient Health Questionnaire. *JAMA*, *282*(18), 1737-1744. <https://doi.org/10.1001/jama.282.18.1737>

United Nations High Commissioner for Refugees [UNHCR]. (2024). *Global trends: Forced displacement in 2023*. UNHCR. <https://www.unhcr.org/global-trends-report-2023>

van Reisen, M., Al-Qasim, T., Christophe, F., Estefanos, M., Z., G., E., G., Höfner, S., Kidane, S., Kuilman, S., Mekonnen, D., Mawere, M., Plaut, M., Reim, L., Sereke, W., Smits, K., & Tewolde, Z. (2017). *Human trafficking and trauma in the digital era: The ongoing tragedy of the trade in refugees from Eritrea*. Langaa RPCIG.

Yuval, K., Aizik-Reebs, A., Lurie, I., Demoz, D., & Bernstein, A. (2021). A functional network perspective on posttraumatic stress in refugees: Implications for theory, classification, assessment, and intervention. *Transcult Psychiatry*, *58*(2), 268-282. <https://doi.org/10.1177/1363461520965436>

Yuval, K., & Bernstein, A. (2017). Avoidance in posttraumatic stress among refugee survivors of violent conflict and atrocities: Testing trans-cultural risk processes and candidate intervention targets. *Behav Res Ther*, *99*, 157-163. <https://doi.org/10.1016/j.brat.2017.10.008>

Yuval, K., Zvielli, A., & Bernstein, A. (2016). Attentional bias dynamics and posttraumatic stress in survivors of violent conflict and atrocities. *Clinical Psychological Science*, *5*(1), 64-73. <https://doi.org/10.1177/2167702616649349>

**Results Supplemental**

**Table 5**

*Linear Regression of Levels of Prospective Pre-intervention Factors Predicting Relative Percentage of Incomplete Sessions + Practices Levels*

|  | Relative Percentage of Incomplete Session Modules | | | | | Relative Percentage of Incomplete Meditation Practice Exercises | | | | |
| --- | --- | --- | --- | --- | --- | --- | --- | --- | --- | --- |
| **Predictors** | *F* | *df* | *β* | *p* | *R²* | *F* | *df* | *β* | *p* | *R²* |
| Sex assigned at birth | 3.50 | 55 | 0.24 | 0.07 | 0.06 | 0.87 | 49 | 0.13 | 0.36 | 0.02 |
| Post-Migration Living Difficulties (PMLD) | 1.47 | 54 | 0.16 | 0.23 | 0.03 | 0.77 | 49 | 0.12 | 0.39 | 0.02 |
| COVID-19-Related Socioeconomic Insecurity | 2.52 | 55 | 0.21 | 0.12 | 0.04 | 2.81 | 49 | 0.23 | 0.10 | 0.05 |
| Post-traumatic Stress Symptoms (HTQ-5) | 1.46 | 53 | 0.16 | 0.23 | 0.03 | 2.91 | 48 | 0.24 | 0.09 | 0.06 |
| Depression symptoms (PHQ-9) | 0.01 | 54 | 0.01 | 0.94 | 0.00 | 0.72 | 48 | 0.12 | 0.40 | 0.01 |
| Anxiety Symptoms (BAI) | 0.37 | 54 | 0.08 | 0.55 | 0.01 | 1.28 | 48 | 0.16 | 0.26 | 0.03 |

|  |  |  |  |  |  |
| --- | --- | --- | --- | --- | --- |

*Note.* Asterisks *, **, and *** represent significance levels at 0.05, 0.01, and 0.001, respectively.

PMLD = Post-Migration Living Difficulties scale (Silove et al., 1997); HTQ-5 = Harvard Trauma Questionnaire-5 (Berthold et al., 2019); PHQ-9 = Nine-item Patient Health Questionnaire (Spitzer et al., 1999); BAI = A brief version of the Beck Anxiety Inventory (Beck et al., 1988).

**Table 6**

*Linear Regression of Levels of Relative Percentage of Incomplete Sessions + Practices Predicting Levels of Residualized Change Scores of Stress- and Trauma-Related Mental Health Outcomes*

|  | Relative Percentage of Incomplete Session Modules | | | | | Relative Percentage of Incomplete Meditation Practice Exercises | | | | |
| --- | --- | --- | --- | --- | --- | --- | --- | --- | --- | --- |
| **Predictors** | *F* | *df* | *β* | *p* | *R²* | *F* | *df* | *β* | *p* | *R²* |
| Post-traumatic Stress Symptoms (HTQ-5) | 0.00 | 52 | 0.00 | 0.99 | 0.00 | 0.62 | 47 | -0.11 | 0.44 | 0.01 |
| Depression symptoms (PHQ-9) | 0.46 | 53 | 0.09 | 0.50 | 0.01 | 0.37 | 47 | 0.09 | 0.55 | 0.01 |
| Anxiety Symptoms (BAI) | 1.57 | 53 | 0.17 | 0.22 | 0.03 | 0.50 | 47 | 0.10 | 0.48 | 0.01 |

*Note.* Asterisks *, **, and *** represent significance levels at 0.05, 0.01, and 0.001, respectively.

HTQ-5 = Harvard Trauma Questionnaire-5 (Berthold et al., 2019); PHQ-9 = nine-item Patient Health Questionnaire (Spitzer et al., 1999); BAI = A brief version of the Beck Anxiety Inventory (Beck et al., 1988).

**Excluded (*n* = 30)**

**Not meeting inclusion criteria (*n* = 6)**

Fluent and literate in Tigrinya (*n* = 5)

Own a personal smartphone (*n* = 1)

**Meeting exclusion criteria (*n* = 5)**

Current mental health treatment (*n* = 3)

Participation in a past Mindfulness-Based Trauma

Recovery for Refugees intervention study (*n* = 2)

**Declined to participate (*n* = 19)**

**Assessed for eligibility** **in phone interview (*n* = 96)**

**Consenting participants that started pre-intervention assessment (*n* = 66)**

**Eligible and consenting participants that completed pre-intervention assessment (*n* = 60)**

**Received at least one session module**

**of allocated intervention program** **(*n* = 60)**

**Completed post-intervention assessment** **(*n* = 59)**

**Did not complete post-assessment (*n* = 1)**

Lost contact (*n* = 1)

**Excluded (*n* = 6)**

**Meeting exclusion criteria (*n* = 5)**

Active suicidality (*n* = 5)

**Declined to participate (*n* = 1)**

**Fig. 1**

*Nonrandomized, Single-Arm, Open-Trial Mindfulness-SOS for Refugees*

*Consort Diagram*

**Analysed**

Full case complete Intent-to-Treat analysis (*n* = 60)

Sensitivity analysis excluding two outliers on utilization engagement (*n* = 58)
